# Self-organisation in striped seagrass meadows affects the distributional pattern of the sessile bivalve *Pinna nobilis*

**DOI:** 10.1038/s41598-019-43214-6

**Published:** 2019-05-10

**Authors:** Stefania Coppa, Giovanni Quattrocchi, Andrea Cucco, Giuseppe Andrea de Lucia, Sara Vencato, Andrea Camedda, Paolo Domenici, Alessandro Conforti, Andrea Satta, Renato Tonielli, Monica Bressan, Giorgio Massaro, Giovanni De Falco

**Affiliations:** 1 CNR - Consiglio Nazionale delle Ricerche, IAS - Istituto per lo studio degli impatti Antropici e Sostenibilità in ambiente marino, Località Sa Mardini, Torregrande, Oristano Italy; 2 Università di Padova, Dipartimento di Biologia, Padova, Italy; 3 CNR - Consiglio Nazionale delle Ricerche, ISMAR - Istituto di Scienze Marine, Napoli, Italy; 4 Area marina protetta “Penisola del Sinis-Isola di Mal di Ventre”, Cabras, Italy

**Keywords:** Ecology, Earth and environmental sciences

## Abstract

Striped seagrass meadows are formed by narrow ribbons which are elevated over the seabed and separated by channels. Limited information on the genesis and development of this morphological pattern, including the adaptive responses of associated biota, is preventing holistic insight into the functioning of such protected ecosystems. This paper assessed the structural dynamics of a *Posidonia oceanica* striped meadow and the distribution and 3D orientation of the associated bivalve *Pinna nobilis*. Our analysis of the interaction between bedforms, bottom currents, and the distribution of *P. nobilis* revealed that the striped seascape is the result of a self-organisation process driven by feedback interactions among seagrass growth, sediment deposition, and hydrodynamics. The results suggest that the ribbon wall is the most suitable sub-habitat for this species, because it supports the highest density of P. nobilis, compared to the meadow top and bottom. Here, specimens can take advantage of the resuspension induced by hydrodynamics and open their shells towards the current, thus enhancing food intake. Therefore, our results show that self-organisation in striped seagrass meadow affects the distributional pattern of *P. nobilis,* providing new insights into the autoecology of this species beyond the conservation implications for its habitat.

## Introduction

The role of engineer species in modifying sediment dynamics and landform processes has recently caught the interest of the scientific community with respect to biogeomorphology e.g.^1,2^. Currently, biogeomorphology research is focused on water sediment-driven systems in coastal areas where plant species play a key role in shaping the ecosystem e.g.^3–5^.

Seagrasses are rhizomatous angiosperms forming highly structured meadows. They are one of the most complex landscapes common in littoral ecosystems^6^. Seagrasses typically grow between 0 and 30 m deep, where abiotic (mainly wave action, currents, sediment input, and light) and biotic factors (interspecific competition and grazing) including anthropogenic stressors (e.g., pollution, trawling) can limit their spatial spreading and structure e.g.^7–12^. The recent literature has demonstrated that the structure of a meadow can be the result of a feedback interaction between seagrass and the surrounding environment, rather than the multiple actions of physical stressors^13,14^. For instance, seagrasses can enhance their own growth by reducing local hydrodynamic activity and resuspension phenomena^14^, thus modifying ecosystem processes and landscapes as all geomorphologic/engineer species do^1^. Besides contributing to the physical equilibrium of coastal areas, seagrass meadows are among the most productive ecosystems in the world and serve as a key habitat in the life cycles of many endangered and commercial species^15,16^. In recent decades, advances in remote sensing/hydro-acoustic technology and geographic information systems have contributed greatly to the study of seagrasses, allowing us to assess their geographical distribution, meadow spatial patterns and conservation trends.

Whilst progress has clearly been made on understanding the interactions between seagrasses and the physical environment, many gaps still remain in our knowledge of the biogeomorphology of these meadows^5^, such as a definition of the processes and dynamics determining different ecomorphoses (i.e. particular meadow morphology linked to local ecological conditions: mosaic-shaped, barrier reef, striped and hill-shaped). Furthermore, the adaptive responses to the meadow spatial pattern by the associated biota are largely unknown^17,18^. Our current work aimed at investigating the relationship between the morphodynamics of a *Posidonia oceanica* (L. Delile, 1813) striped meadow and the distribution of the associated sessile bivalve *Pinna nobilis* (L., 1758). Both of these endemic Mediterranean species are particularly affected by anthropogenic impacts and are therefore protected under the Habitats Directive (*P. nobilis* is listed in Annex IV and *P. oceanica* is listed in Annex I), the Berne Convention (*P. oceanica*, Annex II), and the Protocol on Mediterranean Specially Protected Areas and biological diversity in the Mediterranean of the Barcelona Convention (both are listed in Annex II). Although the ecomorphosis of the striped meadow is included in the above protocol as a priority habitat for conservation (ID code: III.5.1.1), little information is available on its geographical distribution, and the dynamics promoting its structural development have not been demonstrated yet.

*Posidonia oceanica* can display a striped pattern in shallow areas (0.5–3 m in depth), appearing as long, narrow ribbons alternating with channels of dead *matte* or bare sand^19–21^. The former case was described by Boudouresque *et al*.^19^. which suggested that ribbons continuously shift at a speed of about 10 cm/year as they are forced by a unidirectional current flowing in the opposite direction. According to this hypothesis, every ribbon should have an asymmetric section with an accumulation side and an erosive wall. The authors did not give an explanation of ribbon formation. The latter case, where ribbons alternated to bare sand, was described by Gobert *et al*.^12^, which measured the rate of ribbon migration. They found erosion values for sand corridors in a *P. oceanica* seascape of 0.6–15 cm per year. Striped patterns have also been described for other seagrass species such as *Cymodocea nodosa*^22^ and *Zostera noltii*^14^: the migration of submerged dunes induced by hydrodynamics and self-organisation are the two processes proposed as explanations for these rhythmic structures.

Seagrass meadows are the preferred habitat of the fan mussel *P. nobilis*; the association between this species and *P. oceanica* is stronger in shallow exposed sites where leaves play a key role in attenuating hydrodynamic stress^23–25^. *Pinna nobilis*, with a maximum length of 120 cm and a life span up to 50 years^26^, is the largest long-lived bivalve in the Mediterranean Sea and one of the biggest seashells in the world^27^. As a semi-infaunal suspension feeder, *P. nobilis* lives partially buried in the sediment, and its shell stability is assured by byssus filaments anchored to rhizomes or other solid structures found in the substrate. The populations that have been studied in the Mediterranean Sea show highly variable densities as well as significant differences among habitats, depth ranges, and ecoregions^28^. Aggregated distribution of individuals is common, and this is ascribed to food and habitat availability^29–31^. Preferential shell orientation has been recorded where this is necessary to minimise the drag force^32^ and/or to maximise food intake^33^ to enhance individual survival.

**Figure 1:**
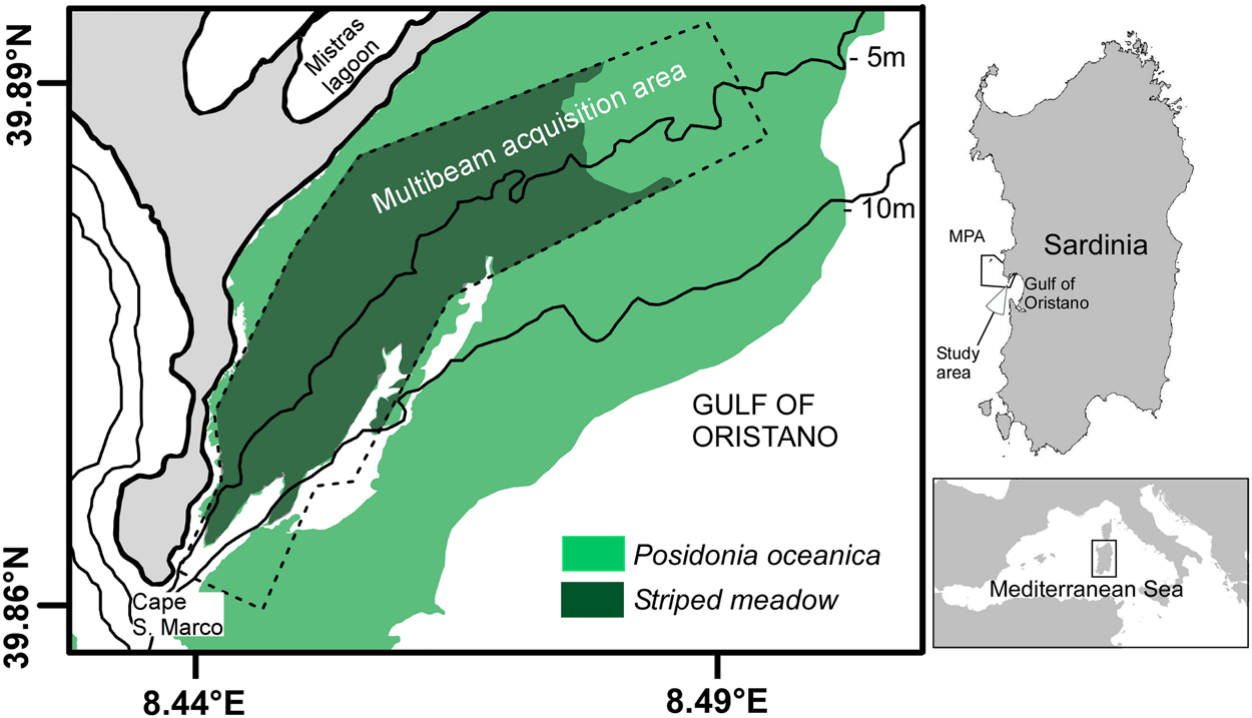
Map of the study area showing the distribution of *Posidonia oceanica* meadows in the northern sector of the Gulf of Oristano, the MultiBeam EchoSounder data acquisition area, and the sector characterised by the presence of the striped meadow.

**Figure 2:**
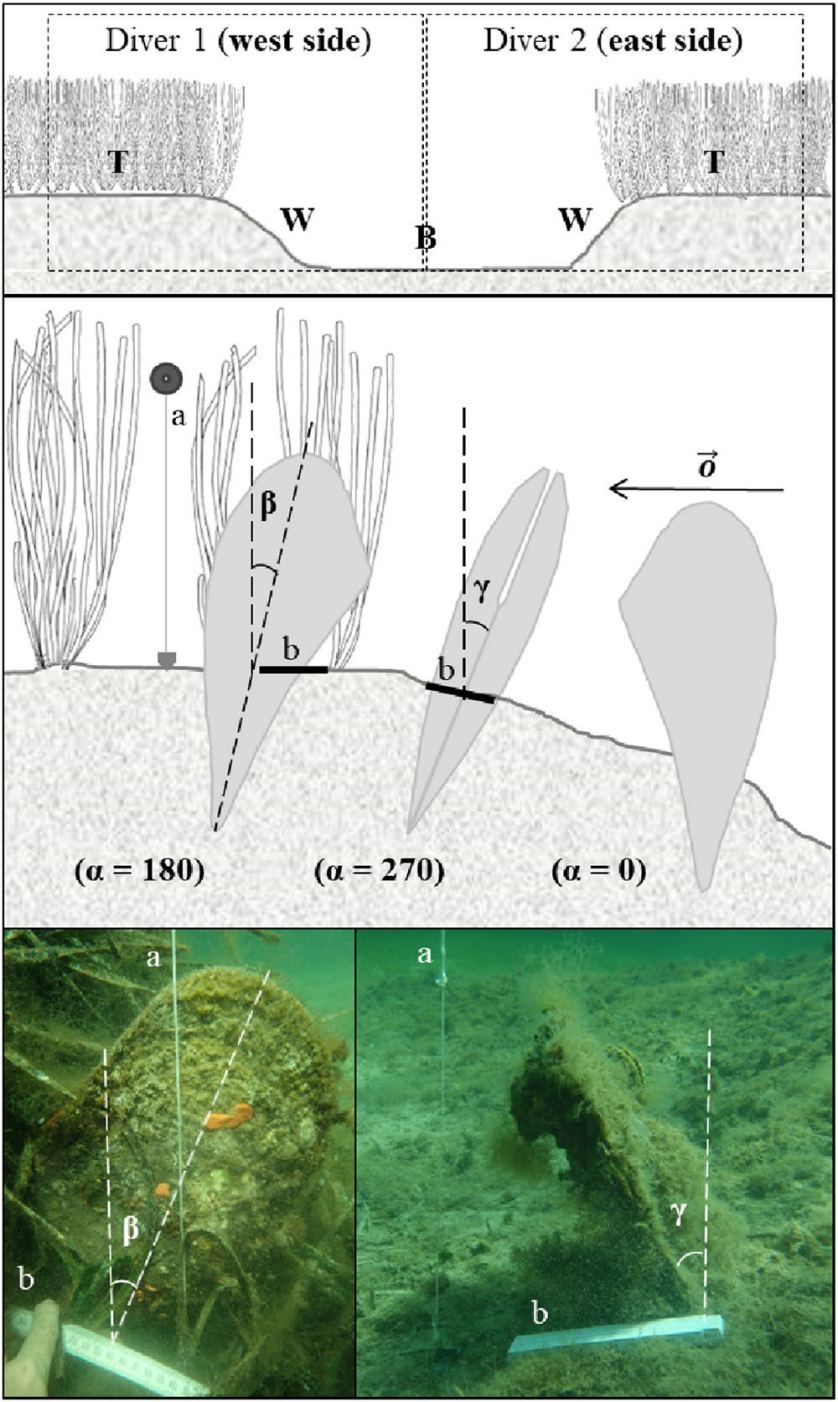
Diagram of the channel section (top; T: meadow top, W: channel wall, B: channel bottom), the measured angles (middle), and sample field images (bottom). α: hinge orientation with respect to the channel slope; β: dorsal-ventral inclination; γ: lateral inclination; a: vertical axis identified by a leaded rope with a small buoy on the top.

This study aimed to assess the process that shapes spatially rhythmic features in *P. oceanica* striped meadows (mobile bedforms *vs*. a self-organisation pattern) and to evaluate the role of biogeomorphological and hydrodynamic factors in defining the distribution pattern for *P. nobilis*. To address these goals, three successive steps were taken: (i) an analysis of a high-resolution digital terrain model (DTM, Fig. 1) of the seabed to define the meadow’s three-dimensional structure and an assessment of the variability of the sediment grain size along meadow cross sections. Subsequently, (ii) the interaction between local hydrodynamics and bedforms was analysed by setting up a three-dimensional non-hydrostatic numerical model and three different simulation scenarios to explain the variability of the flow field acting on the study area. Finally, (iii) the interaction between *P. nobilis*, meadow structure, and hydrodynamics was investigated. Differences in density, mean size, and individual orientation between ribbon sides (accumulation side *vs*. erosive wall) were hypothesized to test whether the bedforms are mobile structures. These population features were compared by meadow layers (top of the ribbons, ribbon walls, and channel bottom; Fig. 2) to test the potential role of the substrate slope, the local currents, and resuspension phenomena in explaining the observed distribution pattern for *P. nobilis*. This paper provides new, fundamental information on the dynamics formation of spatially rhythmic features in seagrass meadows and their relevance for the ecology of *P. oceanica* and *P. nobilis* which should prove useful in improving their conservation.

## Methods

### Study area

The Gulf of Oristano, located on the western coast of Sardinia (Italy), is a semi-enclosed basin of about 150 km^2^ (Fig. 1) with a 9-km-wide opening into the open sea. Except for the two rocky capes, the surrounding coast of the Gulf is sandy and characterised by the presence of various lagoon systems, with the Cabras and Mistras lagoons in the northern side. The Gulf has a soft, slightly sloped bottom (15 m deep in the middle), 70% of which is overgrown by *P. oceanica*^34^. The study area is located in the north-western part of the Gulf (Fig. 1) and consists of a wide striped meadow with a surface of about 4 km^2^ where the ribbons of *P. oceanica* are well defined and the density of *P. nobilis* is high (>2 ind./100 m^2^ according to Coppa *et al*.^33^). This entire area is inside the *Penisola del Sinis – Isola di Mal di Ventre* marine protected area (MPA).

The hydrodynamics in the Gulf are mainly driven by the winds, particularly by the Mistral, which is the dominant wind blowing from the northwest (315°); tides and pressure forcings generated by horizontal water density gradients have no significant effect on the local water circulation^35^. The general circulation pattern is characterised by a clockwise motion, with the sea-water entering the Gulf in proximity to the northern cape, filling the basin, and exiting near the southern cape^35^.

In the study area, the interaction between the Mistral wind and the local basin geometry gives rise to a complex current pattern (see Supplementary Fig. S1). The depth-averaged flow (see Supplementary Fig. S1) is characterised by a counter-clockwise circulation cell whose northern branches move the water masses south-westward along the shore^35,36^. Considering the vertical variability of the flow, the wind’s action generates two different hydrodynamic patterns: a surface one characterised by water currents generally directed off-shore, dragging the surface water masses from the shore towards the Gulf’s centre (see Supplementary Fig. S1); and a bottom current directed cross-shore, replacing the surface waters dragged by the wind with bottom waters (see Supplementary Fig. S1). During Mistral wind events, the intensity of the bottom currents in this area generally varies between 5 and 10 cm/s, as determined by both numerical experiments and direct current measurements^33,35,36^.

### Seabed morphology and sediment sampling and analysis

The morphology of the *P. oceanica* meadow was analysed using Digital Terrain Models (DTMs) derived from MultiBeam EchoSounder (MBES) data, acquired using a Reson Seabat 7125 operating at a sonar frequency of 400 kHz. A total of 6 km^2^ of MBES data were acquired in the northern sector of the Gulf of Oristano, from Cape San Marco and the inlet of the Mistras lagoon in the depth range of 2–15 m (Fig. 1). The bathymetric data were gridded with a grid-cell size of 1 m and a vertical resolution of 0.01 m.

The morphology of the seabed was characterised by the presence of rhythmic features composed of vegetated ribbons and sand corridors. The geometrical characterisation of the seabed was performed using a two-dimensional (2D) spectral analysis in order to identify the sector of the meadow which was characterised by the presence of oriented bedforms^37^. The DTM was divided into 565 200 × 200 m boxes, with an overlap between adjacent boxes of half a box size (see Supplementary Fig. S2). Within each box, a Gaussian window function was applied to minimize the edge effects on the results of spectral analysis. Data were then spectrally transformed using a 2D Fast Fourier Transform (FFT; 1):

1 $$I(\gamma ,\mu )=\frac{{\rm{N}}}{4{{\rm{\pi }}}^{2}}{[\frac{1}{{\rm{N}}}\sum _{{\rm{x}}}\sum _{{\rm{y}}}[{\rm{z}}({\rm{x}},{\rm{y}})-\bar{{\rm{z}}}]{{\rm{e}}}^{-i{\rm{\gamma }}x}{{\rm{e}}}^{-i{\rm{\mu }}y}]}^{2}$$

where z(x, y) is the grid elevation in the x and y directions and $\bar{{\rm{z}}}$ is the average elevation within the box. The 2D FFT matrix was converted from frequencies to wave numbers to scale the frequencies to the distances along the sea floor. The output of the FFT included positive and negative components that mirrored each other. The peaks of spectral strength can be detected in both directions showing the same intensity and opposite angles along the same line (Fig. S2). As a consequence only half the spectrum was used to detect the peaks of spectral strength and, therefore, all the orientations are within the range 0–180°N. Boxes with oriented bedforms produced a clear spectral peak in the direction of the bedform, with a secondary peak related to the bedforms wavelength. Boxes lacking consistently oriented bedforms had a spectrum without any clear peaks. Out of a total of 565 boxes, 151 contained consistently oriented bedforms (see Supplementary Fig. S2). The east and north wave numbers for each of these boxes, were measured at their peak of spectral strength (see Supplementary Fig. S2), to calculate the wavelengths and directions of the bedforms, using the following formulas (2, 3):

2 $$\lambda =\frac{2{\rm{\pi }}}{\sqrt{{{{\rm{k}}}_{{\rm{xm}}}}^{2}+{{{\rm{k}}}_{{\rm{ym}}}}^{2}}}$$

3 $$\phi ={\tan }^{-1}(\frac{{{\rm{k}}}_{{\rm{ym}}}}{{{\rm{k}}}_{{\rm{xm}}}})+{\rm{\pi }}$$

where λ is the wavelength, φ is the direction, and k_xm_ and k_ym_ are the east and north wave numbers that correspond to the peak of spectral strength. Several spectra showed more than one peak related to low frequency components. In this case, we considered only the frequencies, which corresponded to a wavelength between 10 and 40 m, because these frequencies are associated to seabed shape induced by the alternating channels and ribbons of striped meadows.

Bedforms and bottom current orientation were analysed based on circular statistics: to verify whether the mean angles are equal (i.e., in parallel directions, as in mobile bedforms), a circular-circular correlation using a Watson-Williams F-test was performed.

The heights of the bedforms were measured for each selected box from the DTM along profiles transverse to the bedforms (3 profiles, 6 measurements for each profile), and their steepness (height to wavelength ratio) was computed for each box. The spatial variability along the study area of directions, wavelength and steepness of the bedforms was represented using vector and contour maps.

**Figure 3:**
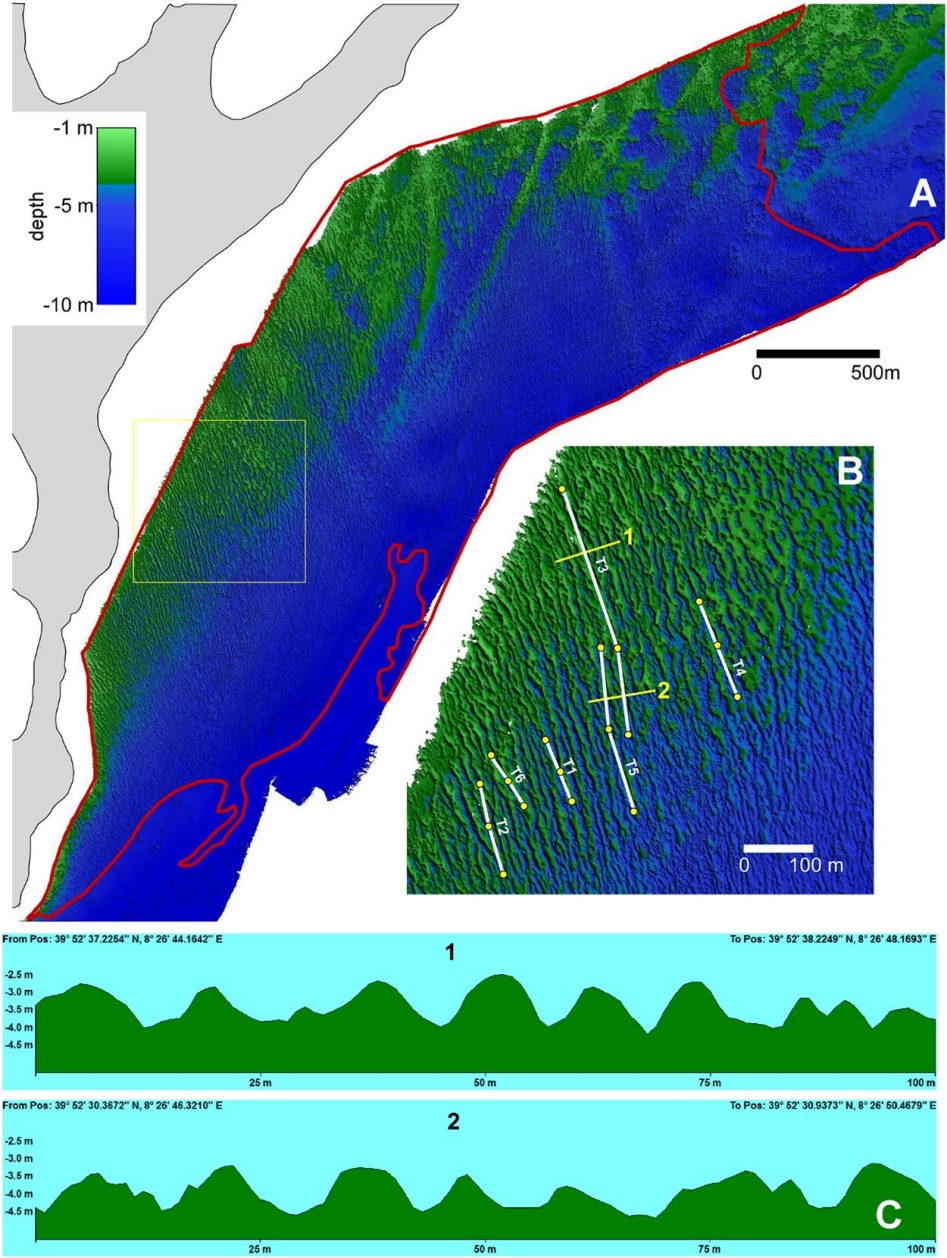
(**A**) Digital Terrain Model (DTM) of the study area: the red line delimits the striped meadow (see Fig. 1); (**B**) DTM sector showing in detail the shape of bedform crests in top view. The channels (T1–T6) and the stations (yellow points) for *Pinna nobilis* distribution analysis and sediment sampling and are also shown. Lines 1 and 2 indicate the locations of the cross sections reported in the lower part of the figure; (**C**) cross section of the bathymetric profiles along the bedform directions.

An area was selected to analyse the relationships between the seabed morphology, the sediment characteristics, and the *P. nobilis* distribution along six transects longitudinal to the sand corridors and ribbon directions (see Figs 1 and 3). The slopes of both sides of the channel flanks (hereafter called flank slopes) were measured to evaluate the symmetry of the bedform cross-sections. For each transect, three cross-sections were randomly positioned, and the flank slope was measured from the DTM for each side (six replicates, n = 108). Global Mapper and Surfer software was used for DTM measurements and spectral analysis.

Sediment samples were collected to analyse the differences in sediment grain size along the channel’s section (Fig. 3). Five sampling points were chosen along each section: the meadow top (T), channel wall east side (W_ES_), channel bottom (B), channel wall west side (W_WS_), and meadow top (T) (Fig. 2). A set of 90 sediment samples over all sample points was collected. In the laboratory, the sediments were treated with H_2_O_2_ in order to remove organic matter. They were then wet-sieved at 63 μm. The finer fraction (<63 μm) was analysed using the Galai CIS 1 laser system. The differences in sand content and flank slope along the channel cross sections were tested using a univariate Permanova (two- and three-way respectively). These tests are based on Euclidean distance; each term is analysed using 9999 random permutations and associated with a Monte Carlo test.

### Numerical model and approach

The interaction between the sea-bottom morphology and the bottom flow field generated by the prevailing wind in the study area was studied using the high-resolution, 3D, non-hydrostatic numerical model named Fluidity-ICOM^38^. The observed bedforms mostly show a regular shape that can be represented by a sinusoidal floor if a simplified model approach is adopted to describe the fluid mechanics of the problem e.g.^39,40^. In a model domain with this kind of geometry, the flow in proximity to the sea floor is affected by both viscous and pressure drag, which may generate downstream recirculation due to confined and persistent pressure gradients^41^. Numerical simulation of water flows over a rippled bottom is commonly adopted in fluid dynamics for the study of many phenomena in natural science and engineering applications (e.g., sediment transport, ecological assessment, maintenance of at-sea structures or artificial channels). The numerical model and approach will be described in detail below.

For this case study application Fluidity-ICOM describes the flow of incompressible viscous fluid driven by a stationary force in three dimensions by solving the system of governing equations (4, 5) that read as:

4 $$\frac{\partial {\boldsymbol{u}}}{\partial t}+{\boldsymbol{u}}\cdot \nabla {\boldsymbol{u}}=-\,\nabla p-\rho g+\nabla \cdot {\boldsymbol{\tau }}$$

5 $$\nabla \cdot {\boldsymbol{u}}=0$$

where ***u**** (u, v, w)* refers to fluid velocity ***u*** in the *x, y*, and *z* directions, *p* is the pressure, $\rho$ is the density, and the viscous terms ${\boldsymbol{\tau }}$ are represented with an isotropic stress term (equal to 10^−3^ m/s^2^). The model accounts for pressure gradients and viscous force terms to reproduce the three-dimensional flow. Finite elements are used for integration in space, whereas the integration in time is provided by an implicit scheme that is unconditionally stable and allows for variable time steps. Anisotropic adaptive re-meshing technology and MPI parallelisation^42^ allowed for an efficient numerical integration of the problem (details on the numerical treatment can be found in^38^).

The domain of integration is a channel crossed by a homogeneous flow, and it is wide enough to avoid the propagation of spurious noise generated by any boundary conditions. The computational mesh, which is three-dimensional and unstructured, is composed of about two million nodes with tetrahedral elements of a maximum side length of one meter (the system uses mesh refining technology). The bottom of the box is a wavy wall with a sinusoidal shape and a homogeneous amplitude/wavelength ratio of 0.1. This value was chosen to mimic the bedform features in the study area, where the ratio ranges between 0.13 and 0.08, as estimated by a morphological analysis of the seabed digital model (see ‘seabed morphology and sediment grain size’ in the Results section).

Figure S3 (see Supplementary Information) shows a y-axis-oriented cross-section of the computational grid in the inner part of the model domain characterised by a wavy-like bottom. The domain has one open boundary where a steady current was defined as the flow condition. A no-slip boundary condition was adopted for the fluid flow at the bottom, and free-stress boundary conditions were used for the lateral side-walls and the far-away top. Thus, the adopted boundary conditions read as ***u****(u, v, w)* = 0 at the floor; *w* = 0 and $\partial u/\partial z=\,\partial v/\partial z=0\,\,$at *z* = 0*; v* = *0* and $\partial u/\partial z=\,\partial w/\partial z=0\,\,$along the lateral x-axis-oriented walls. A stationary velocity ***u****(u, v, 0)* of 0.1 *m/s* was prescribed at the y-axis-oriented wall, and at the opposite far-away side a sponge region was adopted to relax the momentum.

In order to investigate the interaction between local hydrodynamics and bedforms, whose crests are mostly oriented as the Mistral wind-induced current field, three different simulation scenarios were set up. Each one is characterised by current fields imposed at the open boundary with 0.1 m/s of speed intensity and with directions parallel to the wavy bottom main axis, in the first case (*Exp* 1), bent 30° clockwise in the second (*Exp* 2) and 30° counter-clockwise in the third case (*Exp 3*). The 30° deviation was selected as being representative of the variability of both the wind and current directions in the study area. Therefore, a bending of the water current directions different from 0, as reproduced by *Exp 2* and *Exp 3*, corresponds to the most probable scenario. The arrows at the top of Fig. S3 (see Supplementary Information) describe the direction of the inflow for each experiment. Specifically, *Exp* 1 was forced at the open boundary with ***u****(u*_*1*_*, 0, 0)*, *Exp* 2 with ***u****(u*_*2*_*, v*_1_*, 0)*, and *Exp 3* with ***u****(u*_*2*_*, −v*_*1*_*, 0)*, where u_1_ = 0.1 m/s, u_2_ = 0.07 m/s, and v_1_ = 0.03 m/s. Based on the prescribed flow speed (*0.1 m/s*), viscous terms, and channel geometry, the Reynolds Number (*Re*), corresponding to the ratio between inertial and viscous forces, was kept close to the typical values of a transition flow (1000 < *Re* < 2000).

### *Pinna nobilis* sampling and data analysis

The scuba diving fieldwork was performed in the summer of 2016. For the selection of a suitable study area (Figs 1 and 3), a pre-survey was necessary to check the regular pattern of the sea bed and the maintenance of *P. nobilis* density according to the DTM profile analysis and the findings of Coppa *et al*.^33^, respectively. Six channels were then randomly selected (see T1-T6 in Fig. 3), and two divers followed their paths to obtain a measurement of a minimum of 10 *P. nobilis* specimens (depth range 2–5 m). Each diver was assigned half of a longitudinal section of the channel (WS = west side, ES = east side); this included the top of the meadow (T), the wall (W), and half of the sand corridor on the sea bottom (B), each one of these was about 1.5 m wide (Fig. 2). For each *P. nobilis* detected, the minimum width and unburied length were measured using a multi-calliper^43^; the total shell height was estimated using the formula proposed by García-March and Ferrer Ferrer^44^, which is considered universally relevant for the species^45^. The depth, geographical coordinates (see Coppa *et al*.^31^ for more details on methods), position with respect to the channel section (T, W, B), and the distance from the *P. oceanica* edge (positive towards the meadow top and negative going in the channel) were recorded for each individual. The 3D specimen position was assessed by determining the magnitude of the angles α, β, and γ (Fig. 2). The first was measured *in situ* with an underwater compass, considering the vector $\overrightarrow{o}\,$(i.e. half-line indicating the top of the meadow, see Fig. 2) and the channel slope (0° indicates that the dorsal side of the shell is oriented to the top of the meadow; see Fig. 2). A photograph of one frontal and one lateral image per *P. nobilis* (Fig. 2), taken with a high resolution digital camera, allowed the measurement of the angles β and γ using *Golden Ratio* software.

Differences in population density of *P. nobilis* among channels (T1–T6), their longitudinal sections (WS, ES) and meadow layers (T, W, B) were assessed using a three-way Permanova. The channel was considered a random factor, while channel section and meadow layer were fixed; replicates consisted in three 20 × 1.5 m strip transects for each meadow layer (18 transects per channel). The analysis is based on Euclidean distance; each term is tested using 9999 random permutation and associated with a Monte Carlo test.

Shell orientations were analysed based on circular statistics, unless the data were restricted to a range <90°^46^, as was the case for the substrate slope. The shell orientation values (α, β, γ) did not show significant differences among the six channels for either the west or east side (Watson-William F test, p < 0.05 in all case); therefore the samples were pooled in the analysis. Watson U² and Watson-William F tests were applied respectively to check whether the distributions of α, β, and γ were uniform and whether the mean angles differed between the east and west sides. Circular-linear correlations were then performed to test for the presence of any shell orientation (angles α, β, and γ) related to the size of the individuals, their position along the channel side (T, W, and B), or the substrate slope.

## Results

### Seabed morphology and sediment grain size

The Digital Terrain Model of the *P. oceanica* meadow obtained from MBES data is presented in Fig. 3. In the shallower western sector (<5 m), the seabed is largely characterised by the presence of bedforms with clear directional patterns, while in the deeper eastern sector the DTM revealed a terraced or hummocky morphology, without a specific orientation by the morphological features of the seabed (Fig. 3A). In the plan view (Fig. 3B), the bedforms exhibit a linear to sinuous shape in their crest lines, whereas the cross-sections (Fig. 3C) show that the bathymetric profiles exhibit a sinusoidal shape. Our *in situ* observations revealed that the bedforms are related to the meadow seascape, which exhibits the typical features of striped meadows formed by vegetated ribbons, elevated over the seabed, hundreds of meters long, and alternating with channels that are mainly unvegetated.

**Figure 4:**
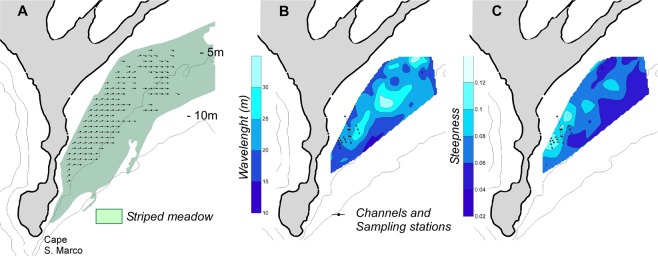
Sector of the seabed with oriented bedforms and spatial variability of their geometrical features as revealed by spectral analysis of the digital terrain model: (**A**) bedform directions, (**B**) wavelength, and (**C**) height-to-wavelength ratio (steepness).

The spectral analysis of the DTM allowed identification of the sector of the seabed where the bedforms show a spatial orientation toward a specific direction (Fig. 4A). This sector is 1.5 km^2^ wide and is located on the western side of the study area. This sector included 151 200 × 200 m boxes, which were used to measure the morphometric characteristics of the bedforms. The direction ranged from 45°N to 110°N (nautical convention) with an average (±SE) of 85 ± 0.9°N. The bedform directions, which are oriented approximately east to west, differed significantly from the dominant current directions at the seabed (Watson-Williams F-test: r = 0.77, p < 0.01), which are oriented approximately south to north (12 ± 1.3°; see Supplementary Fig. S1). The mean wavelength of 21 ± 0.4 m ranged from 10 to 34 m, with the largest bedforms located in the central sector (Fig. 4B). The steepness decreased from west to east (Fig. 4C). In the sampling area, the steepness was in the 0.08–0.12 range, and consequently the average value of 0.1 was used for the 2D hydrodynamic modelling.

The flank slope, measured along the cross-sections of the six transects reported in Fig. 3B, is 22 ± 0.8° and 21 ± 0.9° for the western and eastern channel sides, respectively (see Supplementary Table S1). No significant differences in flank slope were found between the two sides (p = 0.48; see results of 3-way Permanova in Supplementary Table S2), so the sections were substantially symmetrical. Two sedimentary proxies were used to test the difference in sediment grain size along the channel section: sand content and <11 µm content. This latter sediment fraction is known as the ‘non-sortable’ silt fraction^47^, mainly consisting of single particles and an aggregated or flocculated fraction^48^. The sediment was mainly sandy, with differences in sediment grain size along the channel section. The sediment on the bottom of the channel is coarser, with a sand content significantly higher than that found in the channel flanks and the meadow top sectors (see results of the 2-way Permanova and the related pairwise comparison in Supplementary Table S3). On the other hand, the non-sortable fraction (NS) is significantly lower in the channel bottom (see results of the 2-way Permanova and the related pairwise comparison in Supplementary Table S4).

### Hydrodynamics on the wavy floor

**Figure 5:**
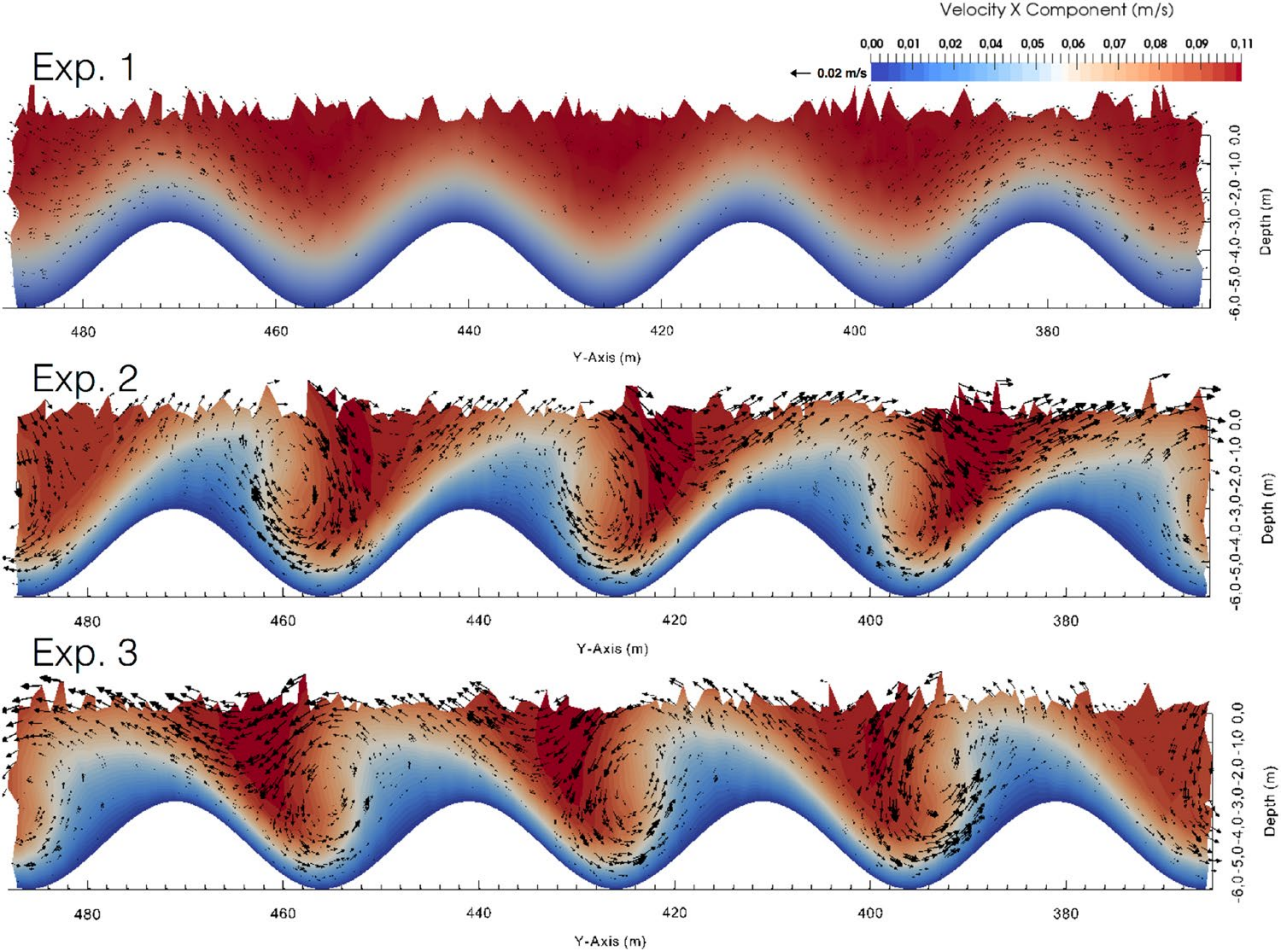
The top, middle and bottom panel refer to *Exp 1, Exp 2* and *Exp 3*, respectively. The colours represent the intensity of the velocity that is normal to the plane of the cross-section (x-axis component) and the arrows represent the intensity of the components that lie on the plane of the cross section (y- and z-axis components).

This section describes the outcomes of the high-resolution hydrodynamic modelling for each scenario (see ‘Numerical model and approach’ in the Methods section). Figure 5 contains a top panel related to *Exp 1*, a middle panel related to *Exp* 2 and a bottom panel related to *Exp* 3. In the pictures, the scaled arrows show the flow speed and direction, ***u****(0, v, w)* on the cross-section plane, and the colours show the intensity of the x-axis-oriented flow, ***u****(u, 0, 0)*. In the *Exp 1* (top panel of Fig. 5), the prescribed flow moves along the wavy bedforms without promoting any significant interaction with the bottom features, as shown by the low intensity of the ***u****(0, v, w)* vector fields. In *Exp 2* and *Exp 3* the prescribed flow is bent 30° clockwise and 30° counter-clockwise, respectively, and promotes recirculation cells along the cross-section plane (see middle and bottom panels of Fig. 5). In both experiments the directions of the ***u****(0, v, w) *components, with maxima of 0.02 m/s, are modulated by the combined effect of both viscous and pressure forcings generated by the interaction of the prescribed flows with the bottom roughness. In both cases, the maximum speed is always found along the x*-*axis, resulting in a three-dimensional flow moving normal to the plane of the section in a helical manner.

**Figure 6:**
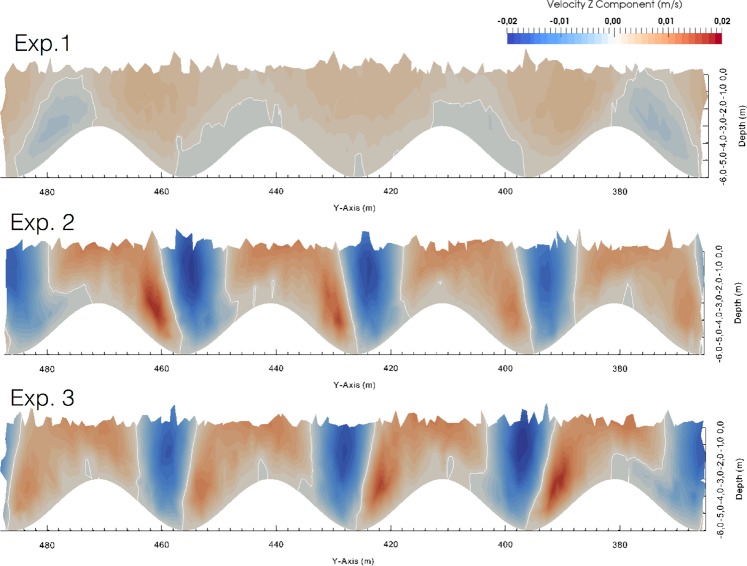
The top, middle, and bottom panel refer to *Exp 1*, *Exp 2* and *Exp 3*, respectively and represent the intensity of the vertical component of the flow; the white contours represent a zero velocity.

The intensity of the velocity component ***u****(0, v, 0)* is mostly below 0.005 m/s. Hence, the dynamic behaviour of the three-dimensional flow is mostly defined by the vertical component of the velocity field. Figure 6 includes a top, middle, and bottom panel for *Exp 1*, *Exp 2*, and *Exp 3*, respectively. In this figure, the white contours indicate zero current speed, and the colour palette indicates the intensity of the vertical component of the current field ***u****(0, 0, w)*. Vertical flows with maxima of 0.02 m/s are found on both sides of the wavy floor slopes, for *Exp 2* and *Exp 3*. Conversely, negligible vertical flows with not ordered features are found in *Exp 1* where the prescribed inflow is parallel to the x-axis.

### *Pinna nobilis* distribution and orientation

A total of 129 *P. nobilis* were recorded in the six channels: 28 were found on the meadow’s top, 83 on the channel wall, and 18 on the bottom. Of the specimens found on the wall, 8% were located on the upper edge (i.e., close to the meadow’s top) and 65% on the lower edge (i.e., next to the bottom). The results of the three-way Permanova showed a homogeneous abundance of *P. nobilis* among channels (p = 0.1341) and their sides (WS = ES: p = 0.4842) as well as a significant difference in individual distribution among meadow layers (i.e., T, W, B: p = 0.0001) (see Supplementary Fig. S4a and Table S5). Post hoc comparisons showed that the density values in the channel wall were significantly higher (see Supplementary Fig. S4a and Table S5) than those recorded at the top of the meadow (p < 0.001) and at the channel bottom (p < 0.01). No difference in *P. nobilis* abundance was found between the T and B layers.

Homogeneity between the channel sides was also demonstrated when considering the size frequency distribution of *P. nobilis*: the profiles obtained for the west and east sides were almost overlapping (Supplementary Fig. S4b; Kolgomorov-Smirnov test: D = 0.1, p > 0.05), showing a unimodal population structure in which the majority of specimens were adults with a size between 55 and 70 cm. The Kolgomorov-Smirnov tests did not find any significant difference in sizes in the comparison of population structures in different meadow layers (Supplementary Fig. S4c; D_T*vs*.W_ = 0.09, D_W*vs*.B_ = 0.10, D_T*vs*.B_ = 0.08, p value always >0.05).

**Figure 7:**
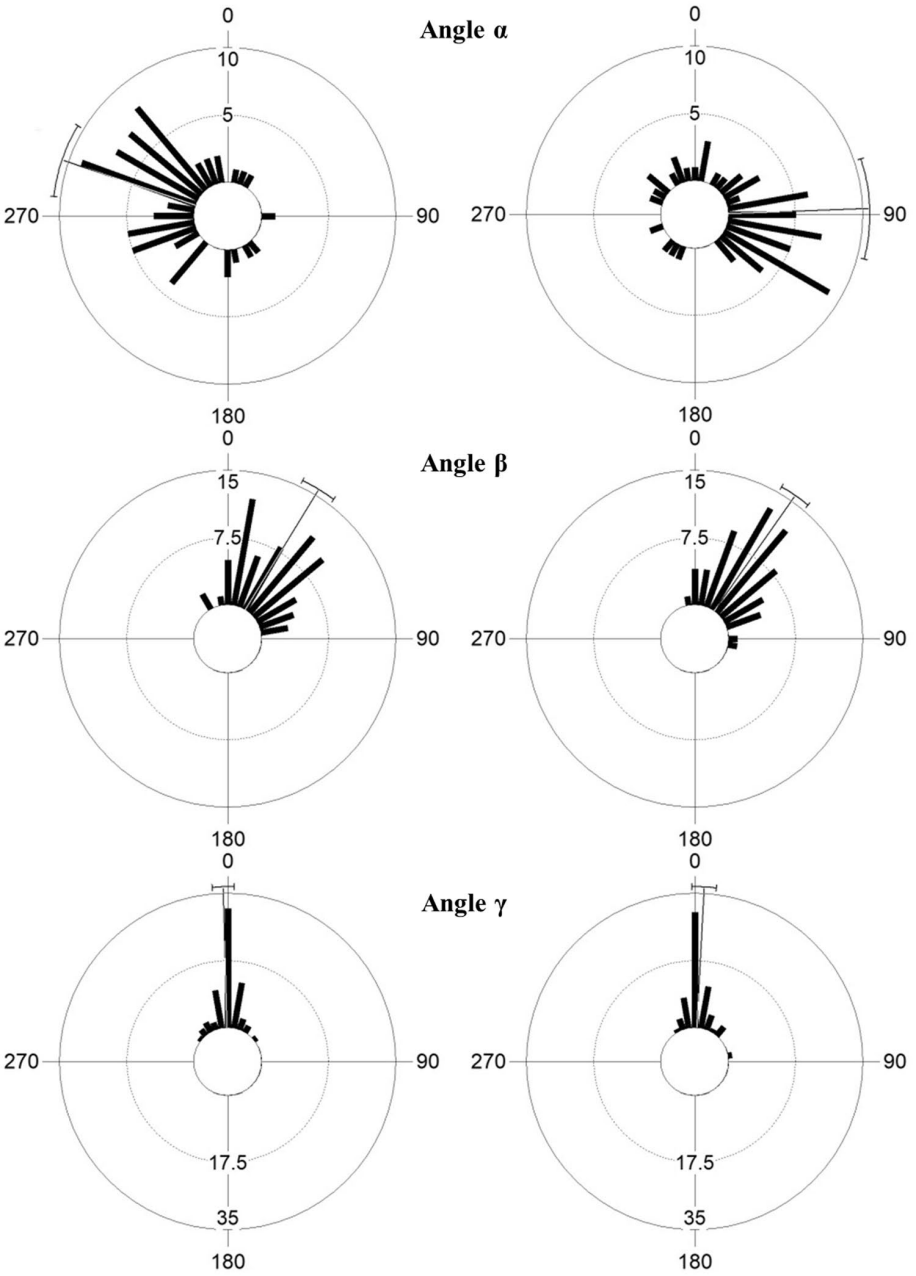
Orientation of *Pinna nobilis* for each side of the channel (west side on the left and east side on the right). Angle α: hinge orientation with respect to substrate slope; Angle β: dorsal-ventral inclination; Angle γ: lateral inclination. The fine line and the whiskers represent the mean and the 95% confidence interval, respectively.

The circular histograms in Fig. 7 show the values of the angles defining the three-dimensional position of *P. nobilis* specimens by channel side. In all cases, the data distribution was clumped (Watson U² test, p value always <0.005).

**Figure 8:**
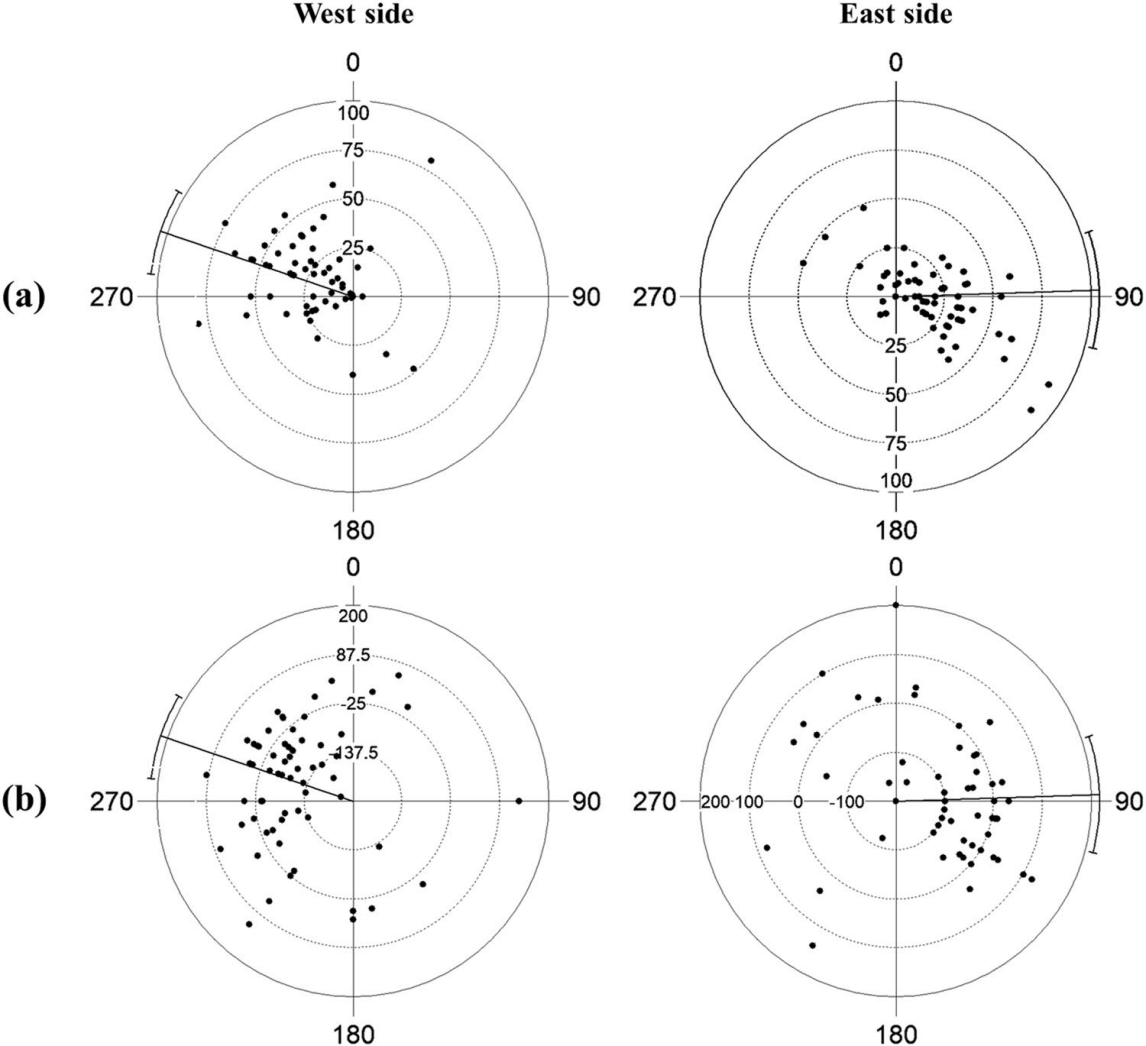
Circular-linear scatter plots of the angle α *vs*. substrate slope (**a**) and *vs*. distance from the *Posidonia oceanica* edge (**b**). Mean and 95% confidence intervals are reported.

The hinge orientation values (α, Fig. 7) showed a mirrored distribution with respect to the longitudinal axis of the channel (Watson-William F test comparing the distribution of ${{\rm{\alpha }}}_{{\rm{W}}S}$ *vs*. $360^\circ -{{\rm{\alpha }}}_{{\rm{ES}}}$; F = 2.642, p > 0.05) with a mean (±SE) of 289 ± 6° (concentration parameter k = 0.672) for the west side and 88 ± 8°(k = 0.553) for the east side. This means that the shell’s opening is towards the bottom of the channel and facing in the direction of the central part of the Oristano Gulf. No significant correlation was found with specimen sizes (WS: r = 0.104, p = 0.526; ES: r = 0.156, p = 0.209), but the variability of α values is related to the substrate slope (WS: r = 0.28, p = 0.01; ES: r = 0.233, p = 0.031) and could be explained by the specimens’ distance from the meadow’s edge (WS: r = 0.296, p = 0.006; ES: r = 0.32, p = 0.001). The circular-linear plots in Fig. 8 show that the specimens found in W or B (negative distance from the edge) had an orientation closer to the mean value than individuals found in the meadow’s top.

No significant difference between the west and east sides was found when considering angle β (Watson-William F test, F = 0.853, p > 0.05), and the pooled sample showed a mean value of 33 ± 2°(k = 0.921) (Fig. 7). The value of angle β was independent from the shell size (r = 0.072; p = 0.525) and the specimen position along the channel profile (r = 0.110, p = 0.219), while a significant circular-linear correlation was demonstrated with the substrate slope (r = 0.675, p < 0.001). Figure S5 (see Supplementary Information) shows the frequency distribution of angle β relating to the vertical axis (a; Fig. 2) and the substrate slope. The majority of the specimens stood up perfectly and were perpendicular to the substrate; the significant difference between the two distributions (Mann-Whitney U-test: U = 667.5, p < 0.0001) revealed a higher relation of angle β to the substrate than to the vertical axis.

The value distribution of angle γ displayed the same pattern (Fig. 7) for the west and east sides (Watson-William F test, F = 2.763, p > 0.05) with a mean value of 0.7 ± 1.4° (pooled sample; k = 0.962). For this variable, no significant circular-linear correlation was found with the size of the individuals (r = 0.060, p = 0.640), substrate slope (r = 0.113, p = 0.197), or the distance from the meadow’s edge (r = 0.033, p = 0.875).

## Discussion

The *P. oceanica* seascape is characterised by architectural and morphological complexity, and a three-dimensional approach is required to study the structure and functional features of the habitat^49^. This is particularly true for striped meadows, where field studies aimed at defining their morphodynamics have not been performed yet, and the influence of this regular pattern on the associated fauna is unknown. The *P. oceanica* meadow analysed in this study can be defined as a striped meadow (*sensu* Boudouresque *et al*.^19,21^) because it is composed of elongated vegetated ribbons separated by channels with a sandy bottom at a depth lower than 5 m. The mechanism of formation and evolution of this bedform, however, is different from those reported by previous studies on striped *P. oceanica* meadows^21^ or sand corridors^12^. The mean bedforms and current directions significantly diverged, forming an average angle of 73 ± 0.8°, and the bedforms did not show differences in flank slope and sediment composition between the sides of the channels. These findings suggest that these spatially rhythmic features are not mobile bedforms over short times, in contrast to the migration rate reported for striped meadows and sand corridors of ~10 cm/year^12,21^.

The spatially rhythmic features that characterised the striped meadows described in this study can be considered to be the results of a self-organisation pattern^14^ driven by the interaction and feedback processes between *P. oceanica*’s growth dynamics, sedimentary processes and bottom hydrodynamics. The concept of self-organisation has been used to explain the formation of geomorphic rhythmic features^50^ and spatial patterns in terrestrial and marine ecosystems^14,51^ that arise from internal organisation caused by the feedback interactions between organisms and their environment^52,53^. The structure of seagrass landscapes may arise from feedback interactions between seagrasses and their environment. It is well established that *P. oceanica* creates a structure (*matte*) consisting of an intertwining of roots, rhizomes, and trapped sediments, which elevates from the seabed. The *matte* structure dampens the currents and waves, buffering fine sediment re-suspension^54^ and enriching *matte* sediments in biogenic carbonate debris^34^.

The morphology of striped *P. oceanica* meadows in this case study can be explained by considering the interaction between meadow growth dynamics, sediment deposition, and the hydrodynamics at the seabed. The output of the Fluidity-ICOM model showed that in proximity to the wavy bottom, representing the morphological features of the striped meadow, the flow moves with a helical behaviour around the slopes of the bedforms. In an experimental setup, Gong *et al*.^55^ reported similar results, with the generation of large stream-wise spiral vortices when oriented flows run over a wavy floor without separation. Barrantes *et al*.^56^ also described the generation of the drag-force induced lateral flow component with non-normal flow incidence. The bedform’s morphology enhances the current velocity inside the channels, thus favouring erosional processes, whereas the current attenuation along the crests enhances fine sediment deposition and vertical growth of the meadow. Consequently, the bedforms can be considered to be the result of a vertical accretion of the *P. oceanica matte* without the horizontal migration induced by currents.

This model is further confirmed by the distribution of *P. nobilis*, which was found on both sides of the channels, without differences in size, density, and orientation between the channel flanks. *P. nobilis* is a sessile, long-living species, large individuals of which could not be present along mobile bedforms. The applied multidisciplinary approach was fundamental in revealing self-organisation as the process driving *P. oceanica*’s biogeomorphology. The analysis of seabed morphology, hydrodynamics, and *P. nobilis* populations with a focus on the interaction between these three elements provided multiple pieces of evidence of the symmetrical structure of the striped meadow, thus refuting the hypothesis of bedform migration.

This study also shows the importance of employing a multidisciplinary approach to the analysis of the population characteristics of associated sessile fauna. In fact, the measurement of habitat spatial patterns is an essential prerequisite to monitoring environmental change and to studying the multi-scale processes that drive the distribution and dynamics of resident species. Many applied environmental disciplines such as conservation, epidemiology, coastal zone management, and ecosystem services analysis should be based on the information from landscape ecology^57^. Despite the proven applicability to marine environments, landscape ecology is not growing as fast as expected, and this is a cause of our limited understanding of species-habitat relationships^58,59^. In a *P. oceanica* seascape, it is known that nutrient load, leaf length, and meadow patchiness could be important factors in defining the occurrence and distribution of associated biota^49^. *Posidonia oceanica* meadows are the preferential habitat of *P. nobilis* due to the physical protection provided by the plant: seagrass leaves attenuate hydrodynamic stresses and hide juveniles from fish predators, and nets of rhizomes allow stronger anchoring than other substrates^23,24,60^. Coppa *et al*.^33^ demonstrated that the distribution of *P. nobilis* and specimen orientation within a meadow were related to the bottom current direction and speed and stated that food availability is likely to be crucial in explaining the observed patterns.

This work has added further knowledge to *P. nobilis*’s autoecology and is the first study aimed at analysing specimen occurrence and orientation in a regularly shaped self-organised seascape. Indeed, as stated by Jackson *et al*.^57^, in analyses of self-organised landscapes, much work has focused on the dynamics of the pattern-forming organism, while the consequences of such regular structures for any associated organisms are poorly understood, including for terrestrial ecosystems. Within the analysed striped meadow, a significantly higher density of *P. nobilis* was found on the channel walls, with four times the values of those recorded on the meadow top and on the channel bottom. In proximity to the sea floor, the transport processes of both inert sediments and nutrients are driven by the interaction between the mechanics of the flow and the specific geometries of the bedforms. The prescribed inflows in *Exp 2* and *Exp 3*, characterised by mirrored orientations, would mimic the real variability of the bottom current in the study area, as it is generated by the mutual interaction of wind, pressure gradients, and morphological-bathymetric features. The resulting clockwise and counter-clockwise helical behaviour of the flow gives us an indication that the slopes of the bedforms are exposed to both upward and downward fluxes with consequent enrichment of the nutrient load, thus making them available to the local sessile fauna. Furthermore it is known that local increases in the flow speed can be found at the edge of the seagrass canopy thus enhancing the nutrients load and the sediments resuspension^61,62^. Hydrodynamics is indeed a fundamental driver in many aspects of benthic biological organisation, including the larval dispersion, settlement, physiology, ethology, and ecology of filter feeders^63^. Here, we infer that the spatial distribution pattern of *P. nobilis*, consisting of a regular alternation between patches with high (on the walls) and low (in T and B) abundance that follow the wavy bedform layout, is strongly related to the self-organised biogeodynamic processes that have modelled the local seascape. This is also supported by the evidence that the abundance of *P. nobilis* progressively increased from the upper edge of the meadow (between T and W) to the boundary with the bottom (W-B; 65% of the sample recorded in W is found on the lower edge), where resuspension is higher. These data are in line with the findings from our first survey^31^ of a wider area of the Gulf, demonstrating that more than half of the recorded individuals colonised the edge of *P. oceanica* patches. Further data would be necessary to assess the food availability among meadow layers and to verify whether the observed distribution is due to different mortality rates and/or to habitat selection by settlers.

Further evidence of the importance of food intake in defining *P. nobilis* spatial distribution was provided when considering the shell orientation (angle α). The majority of the individuals opened their valves towards the channel bottom and in the direction of the middle of the Gulf (i.e., mirrored distribution between channel sides). Therefore, the ventral side of the shells is preferentially oriented toward the bottom current flow (see Supplementary Fig. S1). In a previous study on the influence of hydrodynamics on the same *P. nobilis* population, Coppa *et al*.^33^ found that, on the top of the meadow (*intermatte* channels were excluded from the analysis), bottom currents with an almost constant direction and a speed >0.07 m/s^−1^ can induce specimen directionality with shells aligned and exposed to the flow. The concentration parameter for *P. nobilis* orientation along the channel profiles (k = 0.61) was higher than those found by Coppa *et al*.^33^ on the top of the meadow (k ranges from 0.23 to 0.48); this could be explained by the particular water circulation and resuspension phenomena inside the channels, which offer a greater advantage in terms of growth and survival rates for specimens oriented towards the flow. Combelles *et al*.^64^, analysing a *P. nobilis* population at Port-Cros National Park (France), also found that 80% of the individuals located on a sloping dead *matte* bed were pointing to the bottom. These authors supposed the presence of an ascendant current that favoured more effective capturing of food particles. These studies are in line with the present work that show that the self-organised pattern of the seagrass meadow affects the spatial distribution of *P. nobilis* and the individual orientation demonstrated through its effect on food availability. Feeding is indeed known to be a very important factor in determining the distribution and orientation of many other suspension feeders such as corals, barnacles, crinoids, and mussels e.g.^65–68^.

Another relevant point to stress about the 3D orientation of *P. nobilis* is related to the dorso-ventral and lateral inclination of the shell (angles β and γ). Almost all specimens were located perpendicular to the bottom, revealing a higher relation with the substrate slope than with the vertical axis. In contrast to other species with a byssus apparatus, such as mussels, the large number of filaments (20,000–30,000) and the complex linkages with the rhizome net indicate that the byssus system of *P. nobilis* is adapted for the maintenance of shell position over time^60^. This characteristic is supported by the results of this study, since no differences in dorso-ventral and lateral inclination were observed among sizes or meadow substructures (T, W, B). For these reasons, the initial selection of shell orientation (angle α) and the maintenance of dorsal-ventral and lateral inclinations assured by the byssus filaments must play a fundamental role in assuring individual survival. As mentioned earlier, along the channel profiles, the ventral side of the shells were oriented downwards and the display of such a position with the byssus facing downhill seems to be common on every inclined substrate (Coppa pers. obs.; Combelles *et al*.^64^). It is plausible that this position, in addition to favouring more effective capturing of food particles, may enhance shell stability. A byssus facing downhill can easily keep the shell perpendicular to the substrate through the traction force of the filaments, which tend to pull the shell apex downwards into the sediment and pull the distal side in the opposite direction. The functional dynamics of the byssus and the stage after settlement that allows shell orientation according to hydrodynamics, substrate slope, and matrix should be explored further.

Overall, our results show that self-organisation in striped meadows affects the distributional pattern and 3D orientation of the sessile bivalve *Pinna nobilis*. By assessing how self-organisation shaped the rhythmic features in *P. oceanica* striped meadows and evaluating the influence of habitat structure and hydrodynamics on the distribution pattern of *P. nobilis*, this study adds new ecologically-relevant information on these two protected species. Within the framework of conservation management, besides helping to increase the biodiversity and productivity of coastal environments, the spatial self-organisation enhances ecosystems’ resistance and resilience to disturbance^69^. The increasing anthropogenic impact on the coastal environment (e.g., pollution, dredging, anchoring, illegal trawling) is causing not only seagrass regression but also the modification, or even the loss, of their natural spatial structure^11,49^. Moreover, in the near future, further increases in disturbances as a result of global climate change will need to be faced^69^. For these reasons, assuring the protection of striped meadows as well as any other self-organised ecosystems, along with their processes and functions, should be considered of universal importance. Regarding *P. nobilis*, a strong relationship with the wavy habitat structures and intra-channel hydrodynamics was demonstrated. The walls of the ribbons, supporting a significantly higher density, were proven to be the most suitable sub-habitat for *P. nobilis* within a striped meadow. In this sub-habitat in particular, specimens would take advantage of the resuspension phenomena induced by hydrodynamics and, when located with the shell opening towards the current flow, food capture is likely to be promoted. Whether self-organised habitats promote resident population success in the long run is an open question of fundamental relevance, especially with protected species, and this study contributed to the increasing scientific evidence to help provide proper answers.

## Supplementary information

Supplementary Information

## Data Availability

The datasets generated during and/or analysed during the current study are available from the corresponding author on reasonable request.
