# Supplementary material for: Self-organisation in striped seagrass meadows affects the distributional pattern of the sessile bivalve Pinna nobilis
Source: Sci Rep. 2019 May 10;9:7220. doi: 10.1038/s41598-019-43214-6 (PMC6510729; doi:10.1038/s41598-019-43214-6)
Supplement: Supplementary file 1 — Supplementary Information [file 41598_2019_43214_MOESM1_ESM.docx]

# Supplementary Information for “Self-organisation in striped seagrass meadows affects the distributional pattern of the sessile bivalve *Pinna nobilis*”

Stefania Coppa^1^, Giovanni Quattrocchi^1^, Andrea Cucco^1^, Giuseppe Andrea de Lucia^1*^, Sara Vencato^2^, Andrea Camedda^1^, Paolo Domenici^1^, Alessandro Conforti^1^, Andrea Satta^1^, Renato Tonielli^3^, Monica Bressan^2^, Giorgio Massaro^4^, Giovanni De Falco^1^

^1^CNR - Consiglio Nazionale delle Ricerche, IAS - Istituto per lo studio degli impatti Antropici e Sostenibilità in ambiente marino, Località Sa Mardini, Torregrande, Oristano, Italy

^2^University of Padova, Department of Biology, Padova, Italy

^3^CNR - Consiglio Nazionale delle Ricerche, ISMAR - Istituto di Scienze Marine, Napoli, Italy

^4^Marine protected area “Penisola del Sinis-Isola di Mal di Ventre”, Cabras, Italy

^*^Corresponding author: giuseppe.delucia@cnr.it

**SUPPLEMENTARY FIGURES**


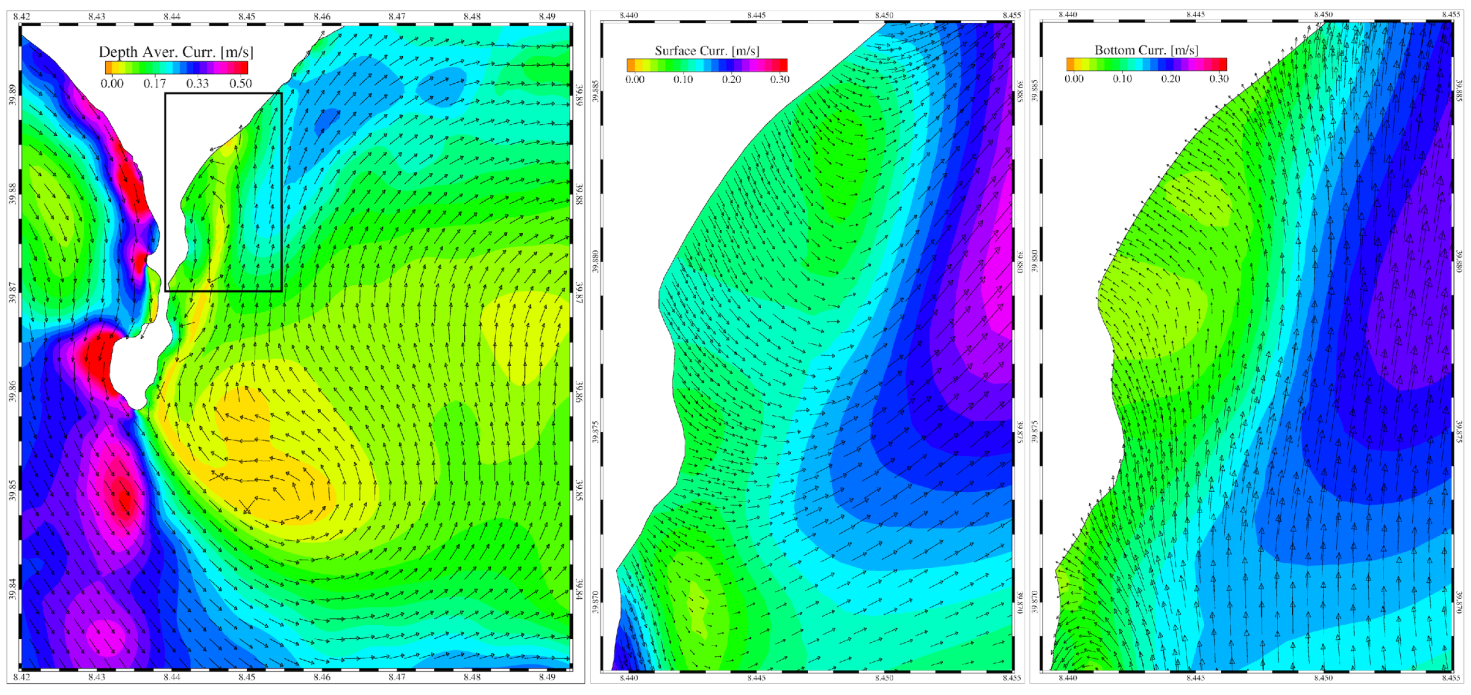


**Fig. S1.** Scheme of the water circulation induced by the Mistral wind. Left panel: Depth-averaged circulation in the northern part of the Gulf and around the San Marco Cape. Black box indicates the site considered. Central and right panels: sea surface (central panel) and sea bottom (right panel) water current fields generated by the Mistral wind within the site considered.


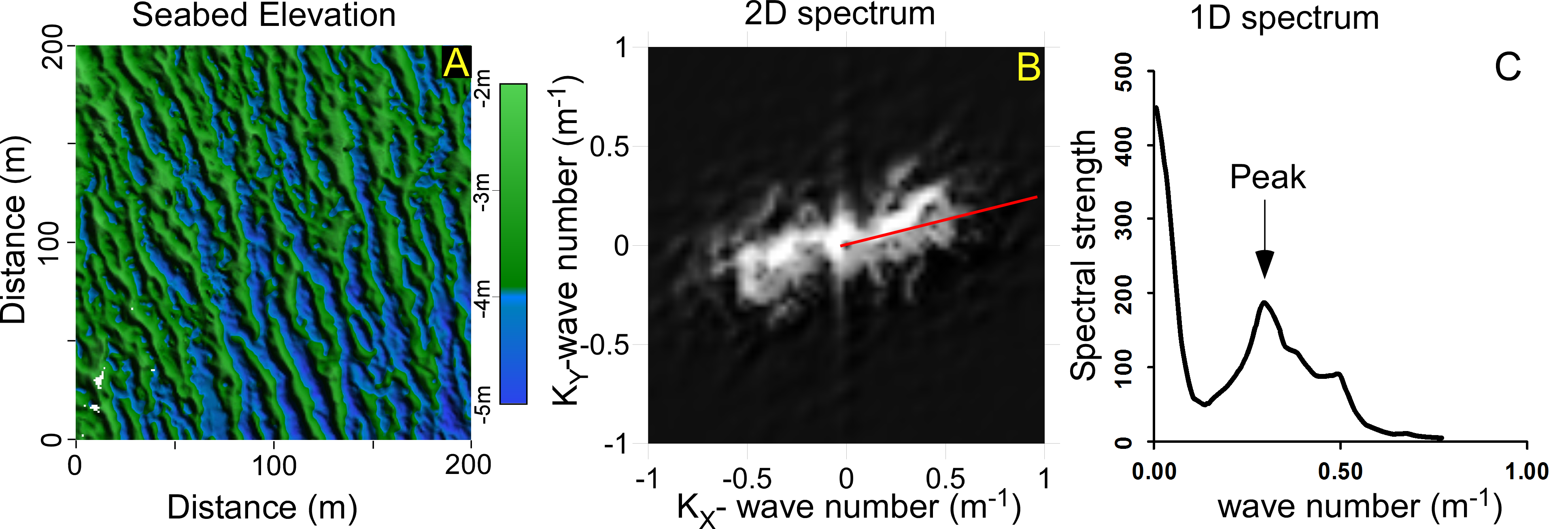


**Fig. S2.** (A) Single square box of the DTM (200 m per side) extracted from the dataset, (B) associated 2D spectrum, and (C) 1D spectrum crossing the peak of spectral strength (red line in B).

*
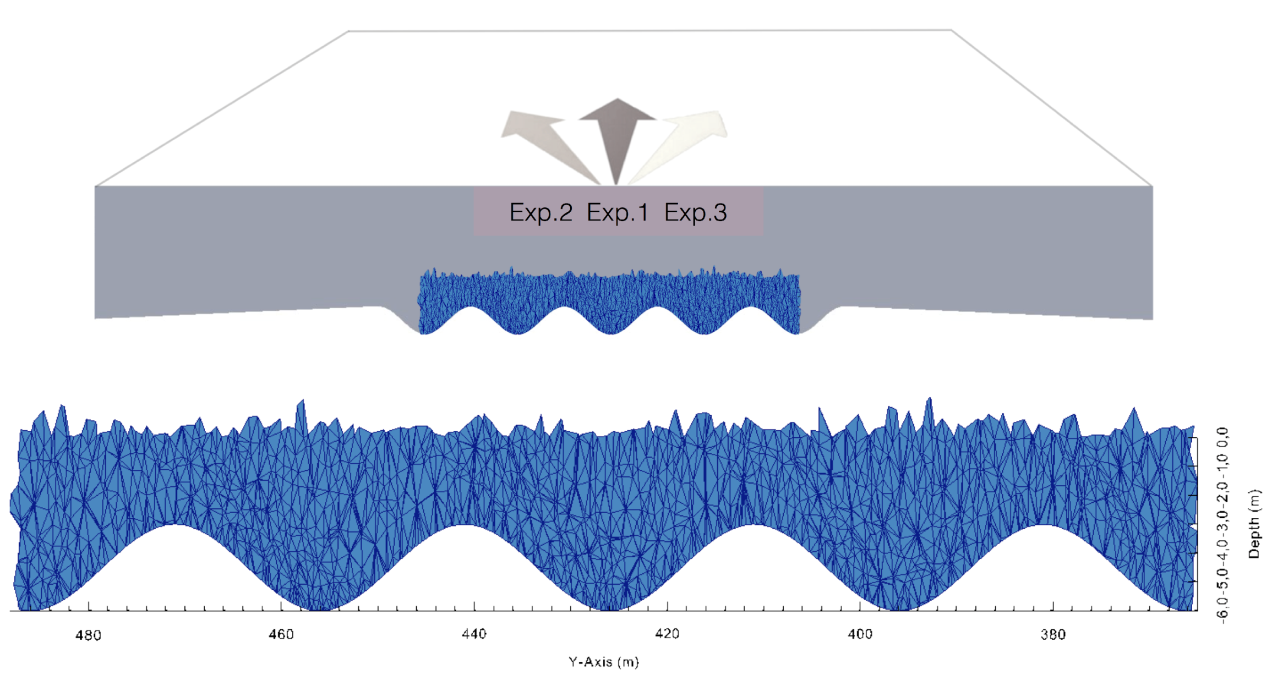
*

**Fig.S3.** Top Panel: Sketch of the domain of integration of the model with inflow direction related to experiments *Exp 1*, *Exp 2*, and *Exp 3*. Bottom Panel: Magnification of a section of the area in proximity to the wavy floor with computational mesh.

**Fig. S4.** (a) Density (mean±SE) and (b) size frequency distribution (%) of *P. nobilis* for each side of the channels and meadow layers (c; top = T, channel wall = W, channel bottom = B).

**Fig. S5.** Frequency distribution of the angle β (20-degree size class, the center of each bin is shown) relative to the substrate slope (light grey) and the vertical axis (dark grey).

**SUPPLEMENTARY TABLES**

**Table S1.** Slope of channel walls and sediment grain size along channel cross-sections.

|  | **Slope (degree)** | | **Sand (%)** | | **<11 µm (%)** | |
| --- | --- | --- | --- | --- | --- | --- |
|  | Mean*±*SE | N | Mean*±*SE | N | Mean*±* SE | N |
| B - Channel bottom | - | - | 98.0±0.2 | 18 | 1.6±0.2 | 18 |
| W - Channel wall (western side) | 22±8 | 108 | 91.7±0.9 | 18 | 6.9±0.7 | 18 |
| W - Channel wall (eastern side) | 21±9 | 108 | 93.2±0.9 | 18 | 5.6±0.7 | 18 |
| T - Meadow top | - | - | 90.2±1.0 | 36 | 7.9±1.1 | 36 |

**Table S2.** Results of three-way Permanova and pairwise comparison on channel slope (S = Shallow; M = middle; D = deep; WS = west side; ES = east side).

|  |  |  |  |  |  |
| --- | --- | --- | --- | --- | --- |
| **Source of variation** | **df** | **MS** | **Pseudo-F** | **p(MC)** | **Pairwise comparison** |
| Channel (1, 2, 3, 4, 5, 6) | 5 | 347.37 | 4.79 | **0.0005** | **Channel** |
| Site (S, M, D) | 2 | 138.09 | 1.91 | 0.1530 | 1≠2 **p<0.05** |
| Side (WS, ES) | 1 | 35.852 | 0.49 | 0.4844 | 2≠3,5 **p<0.001** |
| ChannelxSite | 10 | 72.071 | 0.99 | 0.4549 | 6≠1,3,4,5 **p<0.05** |
| ChannelxSide | 5 | 6.0296 | 0.08 | 0.9949 |  |
| SitexSide | 2 | 70.144 | 0.97 | 0.3831 |  |
| ChannelxSitexSide | 10 | 45.538 | 0.63 | 0.7930 |  |
| Residuals | 180 | 72.457 |  |  |  |
| Total | 215 |  |  |  |  |

**Table S3.** Results of two-way Permanova and pairwise comparison on sand content (T_WS_ = Top, west side; W_WS_ = wall, west side; B = bottom; W_ES_ = wall, east side; T_ES_ = Top, east side).

|  |  |  |  |  |  |
| --- | --- | --- | --- | --- | --- |
| **Source of variation** | **df** | **MS** | **Pseudo-F** | **p(MC)** | **Pairwise comparison** |
| Channel (1, 2, 3, 4, 5, 6) | 5 | 56.94 | 3.11 | **0.0142** | **Channel** |
| Position (T_WS_, W_WS_, B, W_ES_, T_ES_) | 4 | 187.36 | 10.22 | **0.0001** | 1≠2,3,5; 2≠4 **p<0.05** |
| ChxPo | 20 | 14.54 | 0.79 | 0.7142 | **Position** |
| Res | 60 | 18.33 |  |  | B≠ T_WS_, W_WS_, W_ES_, T_ES_ **p<0.001** |
| Total | 89 |  |  |  | T_WS_≠ W_ES_ **p<0.05** |

**Table S4.** Results of two-way Permanova and pairwise comparison on non-sortable content (<11µm) (T_WS_ = Top, west side; W_WS_ = wall, west side; B = bottom; W_ES_ = wall, east side; T_ES_ = Top, east side).

|  |  |  |  |  |  |
| --- | --- | --- | --- | --- | --- |
| **Source of variation** | **df** | **MS** | **Pseudo-F** | **p(MC)** | **Pairwise comparison** |
| Channel (1, 2, 3, 4, 5, 6) | 5 | 144.09 | 2.45 | **0.0463** | **Channel** |
| Position (T_WS_, W_WS_, B, W_ES_, T_ES_) | 4 | 502.79 | 10.70 | **0.0001** | 1≠2,3,5 **p<0.05** |
| ChannelxPosition | 20 | 211.60 | 0.90 | 0.5969 | **Position** |
| Residuals | 60 | 705.10 |  |  | B≠T_WS_, W_WS_, W_ES_, T_ES_ **p<0.001** |
| Total | 89 | 1,563.60 |  |  | T_WS_≠W_ES_ **p<0.05** |

**Table S5.** Results of three-way Permanova and pairwise comparison on *Pinna nobilis* density.

|  |  |  |  |  |  |
| --- | --- | --- | --- | --- | --- |
| **Source of variation** | **df** | **MS** | **Pseudo-F** | **p(MC)** | **Pairwise comparison** |
| Channel (1, 2, 3, 4, 5, 6) | 5 | 0.4941 | 1.7907 | 0.1341 | **Meadow Layer** |
| Side (WS, ES) | 1 | 0.0497 | 0.5623 | 0.4842 | W≠T **p<0.001** |
| Meadow Layer (T, W, B) | 2 | 6.2237 | 39.3730 | **0.0001** | W≠B **p<0.01** |
| ChannelxSide | 5 | 0.0885 | 0.3207 | 0.9004 |  |
| ChannelxLayer | 10 | 0.1581 | 0.5729 | 0.8293 |  |
| SidexLayer | 2 | 0.2643 | 0.5274 | 0.6037 |  |
| ChannelxSidexLayer | 10 | 0.5011 | 1.8163 | 0.0707 |  |
| Residuals | 72 | 0.2759 |  |  |  |
| Total | 107 |  |  |  |  |
